# Effect of Pressure on Thermal Stability of G-Quadruplex DNA and Double-Stranded DNA Structures

**DOI:** 10.3390/molecules181113297

**Published:** 2013-10-29

**Authors:** Shuntaro Takahashi, Naoki Sugimoto

**Affiliations:** 1 Frontier Institute for Biomolecular Engineering Research (FIBER), Konan University, 7-1-20 Minatojima-minamimachi, Chuo-ku, Kobe 650-0047, Japan; E-Mail: shtakaha@center.konan-u.ac.jp; 2 Faculty of Frontiers of Innovative Research in Science and Technology (FIRST), Konan University, 7-1-20 Minatojima-minamimachi, Chuo-ku, Kobe 650-0047, Japan

**Keywords:** DNA, G-quadruplex, high pressure, thermodynamics, volumetric analyses, hydration, molecular crowding

## Abstract

Pressure is a thermodynamic parameter that can induce structural changes in biomolecules due to a volumetric decrease. Although most proteins are denatured by pressure over 100 MPa because they have the large cavities inside their structures, the double-stranded structure of DNA is stabilized or destabilized only marginally depending on the sequence and salt conditions. The thermal stability of the G-quadruplex DNA structure, an important non-canonical structure that likely impacts gene expression in cells, remarkably decreases with increasing pressure. Volumetric analysis revealed that human telomeric DNA changed by more than 50 cm^3^ mol^−1^ during the transition from a random coil to a quadruplex form. This value is approximately ten times larger than that for duplex DNA under similar conditions. The volumetric analysis also suggested that the formation of G-quadruplex DNA involves significant hydration changes. The presence of a cosolute such as poly(ethylene glycol) largely repressed the pressure effect on the stability of G-quadruplex due to alteration in stabilities of the interactions with hydrating water. This review discusses the importance of local perturbations of pressure on DNA structures involved in regulation of gene expression and highlights the potential for application of high-pressure chemistry in nucleic acid-based nanotechnology.

## 1. Introduction

Biomolecules form tertiary structures through noncovalent intra- or intermolecular interactions. These noncovalent interactions are weak compared with covalent bonding and can be easily perturbed by temperature changes. Like temperature, pressure is a key factor of thermodynamics. From a physico-chemical point of view, pressure effects are mainly due to impacts on volumetric aspects of the system. According to Le Chatelier’s principle, the application of pressure shifts an equilibrium toward the state that occupies a smaller volume. Therefore, the properties of biomolecules like volume, compressivity, and expansibility that depend on hydration and molecular packing determine the effect of high pressure on the equilibrium between folded and unfolded states. Pressures for the analysis of biomolecule properties generally range from 0.1 MPa (atmospheric pressure) to 1 GPa. In this range, noncovalent bonding is affected, and high pressure can perturb the tertiary structure of biomolecules and cause the changes in structure or enzymatic activity. The effect of high pressure on protein structures has been relatively well characterized [1,2,3,4]. Most proteins denature or change conformation at high pressure even at low temperature. One can explain the decrease of the partial molar volume of proteins by the penetration of water molecules bound in cavities of structured proteins [5,6,7,8,9,10].

The discovery of high-pressure-induced protein unfolding and denaturation was made in 1914 [11]. It was not until 1964 that the first report of the effect of pressure on a nucleic acid secondary structure appeared [12]. The stability of nucleic acids is determined factors such as base pairing, base stacking, electrostatic interactions, the surrounded solution condition, and so on. Hydration is one of the most important factors to consider. As mentioned above, pressure largely affects the hydration of biomolecules. Analysis under high pressure can provide structural insight into hydrating water. In G-quadruplex formation hydration is a dominant factor for determination of the type of four-stranded conformation and stability. G-quadruplexes as well as other non-canonical structures of DNA (and RNA) can regulate biological processes such as transcription and translation [13,14]. Therefore the pressure effect on these structures is of interest as transient pressure differentials inside living cells might impact the stabilities of these and other non-canonical structure of nucleic acids. Furthermore, the different sensitivity of each DNA structure to pressure is possibly useful to develop nano-materials triggered by pressure effects.

In this review, we focus our attention on the effect of hydrostatic pressure on the stability of nucleic acid structures. First, we discuss previous research into the pressure effect on double-stranded DNA by using thermodynamic, kinetic, and structural analyses. Second, we discuss what is known about the pressure effect on non-canonical structures of nucleic acids, especially the G-quadruplex, and describe how high pressure study of nucleic acids may lead to control of gene expressions of cellular functions, and permit to design novel materials of nucleic acids.

## 2. Pressure Effect on Canonical Duplex of Nucleic Acids

### 2.1. Melting Analysis under High Pressure by Temperature Change

To investigate the structural stability of nucleic acids, temperature change experiments are often used because helixes of DNA and RNA can reversibly unfold upon heating and refold upon cooling. Because of hypochromism, the helix form of nucleic acids has a different ultraviolet (UV) absorption (different molar extinction coefficient) from the random coil form. Analysis of the circular dichroism (CD) spectrum over a range of temperatures is also widely used because CD is highly sensitive to the structural transitions of nucleic acids. Thus, UV and CD melting curves can be used to study the thermal stability of nucleic acids. The temperature at the midpoint of absorbance change is called melting temperature, *T*_m_. In physical terms, *T*_m_ corresponds to the temperature at which ∆*G* = 0 of the equilibrium between folded and unfolded conformations of nucleic acids. When pressure is applied, the equilibrium can shift resulting in either a *T*_m_ increase or decrease. Considering the Clapeyron equation:

d*T*_m_/d*P* = *T*_m_∆*V*_tr_/∆*H* (1)

where the volumetric parameter ∆*V*_tr_ [15] for formation of the folded structure of nucleic acids can be obtained from several series of *T*_m_ measurements at different pressures. To calculate the *∆V*_tr_ value, ∆*H* is required. ∆*H* can be calculated from the helix-coil transitions as the van’t Hoff enthalpy from the optical and spectropic data. The value of ∆*H*_cal_ obtained from calorimetry can also be utilized. When *∆T*_m_/*∆P* is positive and ∆*H* is negative, *∆V*_tr_ must be negative, which means the stability of nucleic acid is promoted with increasing pressure.

In the early studies, the effects of pressure on natural DNAs were investigated. Due to availability, calf thymus DNA has been analyzed intensively to study the thermodynamic parameters with changing pressure. Weida and Gill reported *T*_m_ changes of calf thymus DNA under high pressure followed using CD technique. In the presence of 30 mM NaCl, the value of d*T*_m_/d*P* was 4.49 × 10^−2^ K·MPa^−1^ [15,16]. This corresponds to a *∆V*_tr_ value of −4.5 cm^3^·mol^−1^ (Table 1). Gunter and Gunter carried out similar experiments in the presence of 140 mM KCl and obtained the values of 2.34 × 10^−2^ K·MPa^−1^ and −2.7 cm^3^·mol^−1^ for d*T*_m_/d*P* and *∆V*_tr_, respectively (Table 1) [17]. Nordmeier revealed the dependency of salt concentration on the volumetric parameters [18]. In a series of KCl concentrations, the magnitude of *∆V*_tr_ increased with increasing the salt concentration (Table 1). DNA isolated from *C. perfringens* was examined by Hawley and MacLeod, who showed that the values of ∆*T*_m_/∆*P* were positive and increased linearly with NaCl concentration (Table 1) [19]. Thus, the structure of natural DNA was stabilized by pressure and salt. The properties of natural DNA depended on pressure in the opposite direction; protein structure is generally unfolded by pressure. For example, the −∆*V*_unfolding_ (corresponding to *∆V*_tr_ in this review) of RNase A is 45 cm^3^·mol^−1^ and that for SNase is 80 cm^3^·mol^−1^ [20,21,22]. These results mean that under pressure the volumes of these proteins (including the volume of hydration) become much larger and that their tertiary structures tend to unfold. 

**Figure 1:**
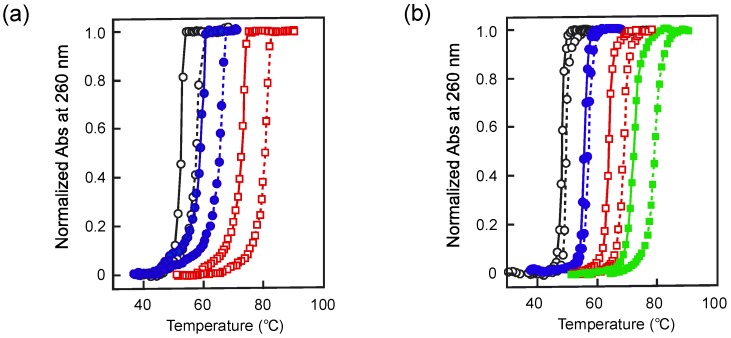
Normalized UV melting curves at different NaCl concentrations; solid lines are data at 0.1 MPa and dashed lines are at 200 MPa [23]. (**a**) Poly(dA)**·**poly(dT): 20 mM (○, black); 50 mM (●, blue); 200 mM (□, red). (**b**) Poly[d-(A-T)]: 20 mM (○, black); 50 mM (●, blue); 200 mM (□, red); 1 M (■, green).

In further investigations, the effect of pressure on nucleic acids of various sequences and lengths were characterized. Macgregor *et al.* intensively investigated the effect of pressure on synthetic nucleic acids by UV melting under high pressure (Figure 1). Poly[d(A-T)] in the presence of 20 mM NaCl showed a positive value of *∆T*_m_/*∆P* and a negative value of *∆V*_tr_ with a similar magnitude to that of calf thymus DNA in the presence of 5 mM KCl (Table 1) [18]. With increasing concentration of NaCl, these parameters linearly increased [23]. Salt concentration had a relatively large effect on *∆T*_m_/*∆P* and *∆V*_tr_ values for poly(dA)**·**poly(dT) [23], suggesting that the hydration of homopolymers differed from that of natural DNA (Table 1). Although poly[d(G-C)] has a very high *T*_m_ value (over 100 °C), the use of high pressure enabled measurement of the ‘real’ *T*_m_ due to the prevention of boiling. In the presence of 52 mM NaCl, the value of *∆T*_m_/*∆P* was 4.8 times larger and the magnitude of ∆*V*_tr_ value was 5.3 times larger than those of poly[d(A-T)] in the presence of 50 mM NaCl (Table 1) [23]. In the presence of 1 M NaCl, however, the changes in these values of poly[d(G-C)] were only 1.7 times larger than those of poly[d(A-T)], which indicated that the salt dependence of *∆V*_tr_ for poly[d(G-C)] is smaller than that for poly[d(A-T)]. The value ln *K*_obs_/ln [cation] is equal to the number of cations taken up during the formation of duplexes, where *K*_obs_ means the observed equilibrium constant for the formation of the duplex [24]. From this result, it was therefore concluded that a GC base pair binds fewer ions during folding than does an AT base pair.

**Table 1:** Pressure effect of melting temperature and volumetric parameters on natural and synthetic DNAs.

DNA	Salt Concentration	∆*T*_m_/∆*P* (10^−2^ K MPa^−1^)	∆*V*_tr_ (cm^3^ mol^−1^)	Ref.
Calf thymus	[NaCl] = 30 mM	4.49	−4.5	[16]
	[KCl] = 140 mM	2.34	−2.7	[17]
	[KCl] = 5 mM	0.46	−0.51	[18]
	[KCl] = 20 mM	1.4	−1.58	
	[KCl] = 50 mM	2.0	−2.27	
	[KCl] = 200 mM	2.9	−3.32	
	[KCl] = 500 mM	3.5	−4.02	
*C. perfringens*	[NaCl] = 10 mM	0.54		[19]
	[NaCl] = 50 mM	2.0		
	[NaCl] = 120 mM	2.6		
	[NaCl] = 360 mM	3.8		
	[NaCl] = 1.08 M	4.1		
	[NaCl] = 3.6 M	4.6		
poly[d(A-T)]	[NaCl] = 20 mM	0.36	−0.36	[23]
	[NaCl] = 50 mM	0.93	−0.90	
	[NaCl] = 200 mM	2.26	−2.14	
	[NaCl] = 1.0 M	3.86	−3.57	
poly(dA) **·**poly(dT)	[NaCl] = 20 mM	2.49	−2.60	[23]
	[NaCl] = 50 mM	3.15	−3.44	
	[NaCl] = 200 mM	3.86	−4.59	
poly[d(G-C)]	[NaCl] = 52 mM	4.51	−4.80	[24]
	[NaCl] = 107 mM	4.79	−5.16	
	[NaCl] = 300 mM	5.01	−5.50	
	[NaCl] = 1.0 M	6.41	−6.03	
poly(rA) **·**poly(rU)	[K^+^] = 50 mM	−1.07	0.96	[25]
poly[d(I-C)]	[NaCl] = 75 mM	0.28	−0.26	[26]
	[NaCl] = 270 mM	1.36	−1.25	
	[NaCl] = 1.0 M	2.64	−2.39	

The RNA duplex with AU base pairs is slightly destabilized upon pressuring. In the presence of 50 mM K^+^, poly(rA)**·**poly(rU) had a *∆T*_m_/*∆P* of −1.07 × 10^−2^ K·MPa^−1^ and a *∆V*_tr_ of 0.96 × 10^−2^ K·MPa^−1^ (Table 1) [25]. Poly[d(I-C)], containing non-canonical base inosine behaves similarly to poly[d(A-T)] with a positive *∆T*_m_/*∆P* and a negative *∆V*_tr_ value (Table 1) [26] although the magnitude of the values are smaller. In contrast, a methylphosphonate oligonucleotide, in which the charged oxygen of the phosphate group is replaced by uncharged methyl group, showed significant increase of *∆T*_m_/*∆P* [26]. These data emphasize that hydrating water has a prominent effect on the transition volume of nucleic acid unfolding processes. ∆*V*_tr_ can be described as follows:

*∆V*_tr_ = *∆V*_M_ + *∆V*_T_ +*∆V*_I_ (2)

where *∆V*_M_ is intrinsic volume change of the DNA, *∆V*_T_ is thermal volume change indicating the change of the void space of the DNA, *∆V*_I_ is interaction volume change (*i.e.*, hydration volume change) [27]. ∆*V*_M_ and ∆*V*_T_ basically depend on the structure of nucleic acids but ∆*V*_I_ is very sensitive to the number and condition of hydration. RNA and modified nucleic acid can be a negative ∆*V*_tr_ because of a different contribution of ∆*V*_I_ from that of DNA.

### 2.2. Effect of High Pressure on the Conformation of a Duplex

**Figure 2:**
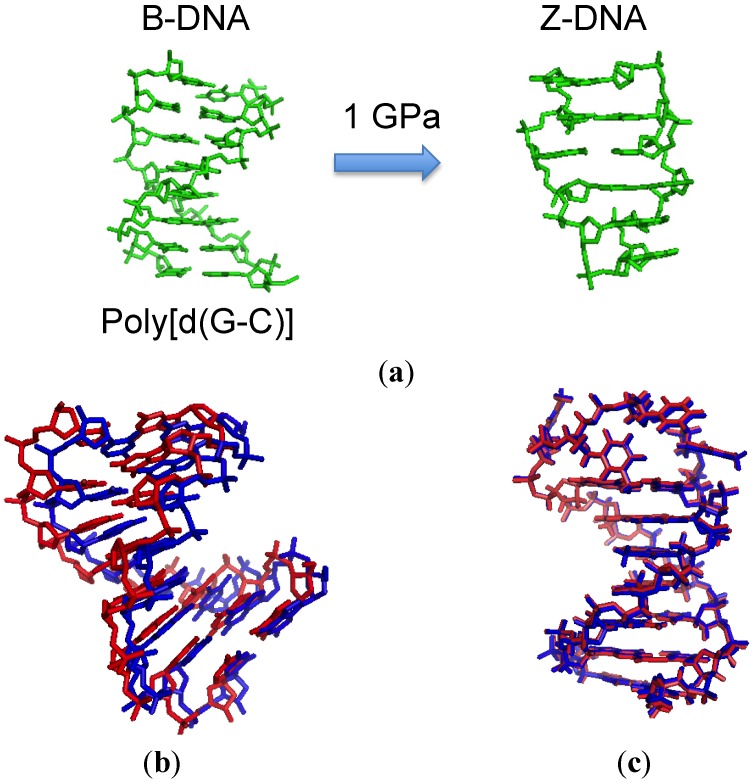
Pressure-induced structural changes to the DNA duplex. (**a**) B-Z transition confirmed by CD spectroscopy; (**b**) Structure of DNA duplex visualized by X-ray crystallography at atmospheric pressure (red) and at 1.39 GPa (blue); (**c**) Structure of DNA hairpin visualized by NMR at 3 MPa (red) and at 200 MPa (blue).

The type of conformation adopted by double-stranded DNA depends on the solvent conditions. B-form DNA changes to A-form in low concentrations of salt or in hydrophobic conditions. The alternate repeat of purine and pyrimidine base pairs forms a left-handed helix, or Z-DNA, in the presence of high concentrations of salt. As shown above, pressure effect on thermodynamics for the nucleic acids largely depends on hydration and salt conditions. This suggests that pressure could induce conformational changes. The B-Z transition was the first of this type of change observed under high pressure (Figure 2a). Kryzyzaniak *et al.* showed that poly[d(G-C)], which is B-form at atmospheric pressure, adopted the Z-form under 1 GPa [28]. They directly monitored the conformational change by using CD spectroscopy with pressuring up to 1 GPa. The conformational changes were monitored by CD spectra which showed a negative Compton effect at 295 nm for Z-DNA. Such a high pressure induces lowering of molecular volume of water from a tetrameric to an octameric form due to shortening of the hydrogen bond (H-bond) distance [29,30]. The structure of water under high pressure resembles that in high salt concentration where Z-DNA is stabilized [31]. This suggested that water under high pressure preferred to interact with phosphate groups of DNA chains located in the groove of Z-DNA. The conformation of the methylated form of poly[d(G-C)] was also investigated under pressure. The transition was not observed under high pressure, although it does occur at atmospheric pressure due to the lower flexibility of methylated nucleotide. In the case of RNA, A-Z transitions of r(GC)_6_ and r(AU)_6_ are observed at 600 MPa in the presence of 5 M NaCl. The concentration of salt required to induce the A-Z transition in RNA is higher than that for poly[d(G-C)], because water binds tightly to the RNA backbone because there are the 2'-OH group [32]. Thus, high pressure can induce Z-form of nucleic acid by the perturbation of the conformation of water molecules, but the flexibility of nucleic acid backbone restricts the effect of high pressure on the structural change. 

A few studies have reported the structure of DNA helix under high pressure determined by using X-ray crystallography or NMR. Girard *et al.* prepared crystals of d(GGTATACC), which forms a self-complementary duplex, and the high-resolution structure was analyzed under 0.55, 1.09, and 1.39 GPa of pressure (Figure 2b) [33]. The crystal structure revealed that the middle of the duplex adopted the A-form, whereas the edges of the duplex formed a disordered B conformation. The spacing of stacked bases shortened by 0.15 Å/GPa and A-DNA hydrogen bonding also shortened by 0.04 Å/GPa. This is in contrast to effects of pressure on bonds in proteins; in proteins, salt bridges and H-bond lengths are usually shortened by ~0.1 Å/GPa [34,35,36]. These differences indicated that the adaptation of DNA to high pressure could be achieved by small variations of arrangements along the backbone. As pressure increases, the first shell of hydration is gradually compressed, but the pentagonal network of water molecules found in major groove is not disrupted. Thus, all the Watson-Crick base pairs and hydrogen networks within major grooves are preserved, which enables the DNA duplex to remain structured at even very high pressure. 

NMR analysis is a powerful technique for study of the effects of pressure on the structure of nucleic acids. NMR under high pressure [37,38,39] was used to investigate the structure of B-DNA (Figure 2c) [40]. The hairpin DNA d(CTAGAGGATCCTUTTGGATCCT) was used, and only the stem region was analyzed. Under 200 MPa, chemical shifts indicated a change in structure of 0.17 Å root mean square relative to the conformation at atmospheric pressure, which is at the lower end of the range of structural changes seen in proteins [37,38,39]. Only 0.042% reduction in volume was observed, corresponding to an intrinsic compressibility of 0.6 × 10^−4^ mL·mol^−1^·bar^−1^ per nucleotide. This value is very small compared to typical adiabatic molar compressibility measured for DNA solutions (30~70 × 10^−4^ mL·mol^−1^·bar^−1^), suggesting that the compressibility of DNA comes from not DNA molecule itself but from the hydration layer surrounding DNA [41]. The biggest change was an increase of the width in the minor groove, suggesting that the hydrating water along the minor groove adopts a different structure with lower partial volume as pressure is increased. In general, the lengths of H-bonds between Watson-Crick base pairs were also reduced. The spacing between AT pairs is 2.6 times more sensitive to the pressure than that of GC pairs. This might be derived from the different numbers of H-bonds in the pairs. The overall length of the stem was slightly increased (1.2%) at high pressure, due to a slight slide of base pairs relative to each other. A structure obtained using X-ray crystallography at high pressure showed a significant reduction in stacking distance. The conflicting results on the effect of base stacking between the crystallography and NMR awaits further investigation. FT-IR technique has also been used to investigate the structural perturbation at high pressure. The IR spectra of poly(dA)·poly(dT) was recorded at 28 °C at up to 1.2 GPa [42]. Although some shifts of prominent band were observed due to the increase of hydration and base stacking, overall the structure was B-form. Therefore, except for the specific sequence under specific conditions, the structure of B-DNA endures perturbation by high pressure. The structure is slightly but certainly affected by pressure: H-bond lengths are shortened and the distance between stacked bases are increased or decreased. The hydration layer is also compressed, and high pressure can induce structural changes to water itself, which better suits the Z-form conformation.

### 2.3. Melting of Duplex Induced by Pressure

The melting and reannealing of duplex nucleic acids is important in reactions in living cells such as replication, transcription, and translation. In nanotechnology, nucleic acid nanodevices are generally based on the control of the stability of duplexes. As shown above, in general DNA polymer duplexes have positive *∆T*_m_/*∆P* and are stabilized under high pressure. If the value of *∆T*_m_/*∆P* is negative, it is possible that applying pressure will induce melting of nucleic acid structure. 

**Figure 3:**
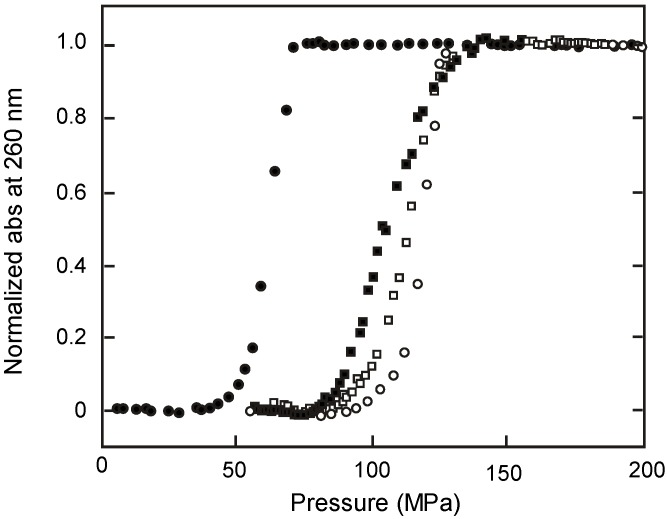
Pressure-induced melting of DNA duplex of poly(dA)**·**poly(rU) at 20 °C (○) and 25 °C (pH 6.7, 28 mM Na^+^) (●); poly(dAdT)**·**poly(dAdT) at 25 °C (pH 6.7, 5.2 mM Na^+^) (■); and poly(dIdC)**·**poly(dIdC) at 25 °C (pH 6.7, 5.2 mM Na^+^) (□) [43,44].

As shown in Table 1, the heteroduplex of poly(dA)**·**poly(rU), a DNA/RNA hybrid, has a negative value of *∆T*_m_/*∆P* in the presence of 50 mM KCl [25]. At neutral pH and in low salt (28 mM Na^+^), this duplex melted sharply with a *T*_m_ of 31 °C under atmospheric pressure [43]. As pressure was increased at 25 °C, the UV absorption at 260 nm of poly(dA)**·**poly(rU) increased beginning at around 50 MPa (Figure 3). At 20 °C, the increase of UV absorption began at about 100 MPa. These results suggest that the profile of the UV absorbance showed a hypochromic effect with increasing pressure due to the induction of the transition of poly(dA)**·**poly(rU) between the duplex and the coil form. Similar results were obtained from the analysis of poly[d(A-T)] and poly[d(I-C)], which showed low *T*_m_ values of 36.0 °C and 29.0 °C at neutral pH in 5.2 mM Na^+^ solution under atmospheric pressure, and could be melted by increasing pressure [44]. Dubins *et al.* [45] simulated the coil-to-helix transition of nucleic acids from the ∆*G*(*P*, *T*) phase diagram; these calculations predicted destabilization of poly(dA)**·**poly(rU), poly[d(A-T)], and poly[d(I-C)] as pressure increased. The melting induced by pressure change is observed only for these specific polymers. For example, the oligonucleotides (dA)_n_(dT)_n_ (where *n* = 11, 15, and 19), which were predicted to be sensitive to pressure melting [46], did not show transitions as pressure was increased. There have been no examples melting of DNA oligonucleotide duplexes by pressure. The existing data do suggest that pressure could affect some reactions of a genomic DNA. For example, the transcription may be started at a region along the genomic DNA partially melted by pressure. Pressure may also be useful in nanomaterials made with nucleic acids. 

### 2.4. Kinetic Analyses

**Figure 4:**
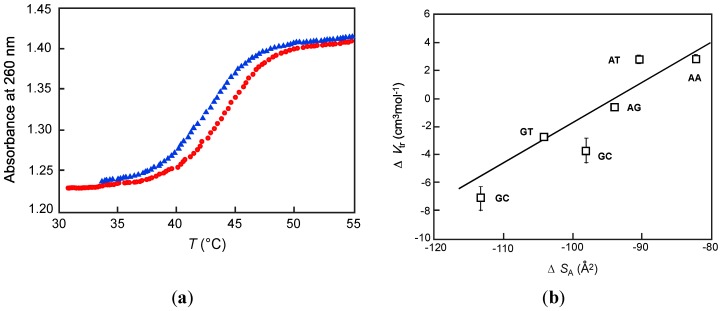
(**a**) UV melting curves of 22-mer DNA used in [43]. The blue triangle and red closed circle represent annealing and melting process, respectively; (**b**) Transition volumes for each of six independent dinucleotide steps plotted as a function of the change in their solvent accessible surface area, ∆*S*_A_ [46].

Kinetic analyses of the coil-to-helix transitions provide informative insights into the mechanism of helix formation and melting regulated with pressure. Analysis of the hysteresis observed during UV annealing and melting processes is convenient for characterization of the kinetic properties of duplex formation (Figure 4a). The forward rate constant *k*_1_ (for the formation reaction) and the reverse rate constant *k*_−1_ (for the melting reaction) can be calculated from the absorbance and temperature changes with time [47]. The rate can be described as:

k = exp{(−∆*V*^‡^/*RT*)*P*} (3)

where *R* is gas constant [46]. By substituting *k*_1_ or *k*_−1_ into Equation (2), the activation volume ∆*V*^‡^_1_ for the forward step or ∆*V*^‡^_−1_ for the reverse step can be obtained, respectively. Upon application of pressure, *k*_1_ becomes larger and *k*_-1_ smaller, resulting in the negative value of ∆*V*^‡^_1_ and positive value of ∆*V*^‡^_−1_, respectively [48,49,50,51]. These results suggested that an increase in base stacking induced by higher pressure accelerated the helix formation. The activation volumes also showed dependency on GC content of the strands. For 22-mer homopurine-homopyrimidine oligonucleotides [48], increasing the fraction of GC base pairs from 0.14 to 0.5 causes ∆*V*^‡^_1_ to increase by a factor of three, whereas the value of *V*^‡^_−1_ became 10 times smaller (Table 2). Furthermore, the subtraction of ∆*V*^‡^_−1_ from ∆*V*^‡^_1_ gives the transition volume ∆*V*_tr kinetic_, which is equivalent to ∆*V*_tr_ obtained from the Clapeyron equation [Equation (1)]. Indeed, volumetric parameters obtained by the two methods are in agreement.

**Table 2:** Activation volume of 22-base duplexes in the presence of 20 mM NaCl [43].

Fraction of GC	∆*V*^‡^_1_ (cm^3^ mol^−1^)	∆*V*^‡^_−1_ (cm^3^·mol^−1^)	∆*V*_tr kinetic_ (cm^3^ mol^−1^) ^a^	∆*V*_tr_ (cm^3^ mol^−1^) ^b^
0.5	−6.7	1.6	−8.3	−5.8
0.32	−8.0	0.40	−8.4	−8.0
0.23	−13	15	−28	−13
0.14	−20	17	−37	−20

^a^ ∆*V*_tr kinetic_ calculated using the activation volumes; ^b^ ∆*V*_tr_ calculated using the Clapeyron equation (Equation (1)). The values given here are estimated to have the errors within ±15%.

Dubins and Macgregor examined the nearest-neighbor effect on the kinetics of the duplex formation under high pressure [51]. The nearest-neighbor model is based on the assumption that the stability of a nucleic acid duplex is determined by type of base pair and the adjacent base pairs, enabling prediction of the thermal stability of a duplex from sequence [52,53,54]. The activation volume (∆*V*^‡^_1_ and ∆*V*^‡^_−1_), the estimated transition volume ∆*V*_tr kinetic_ (=∆*V*^‡^_1_ − ∆*V*^‡^_−1_), and the transition volume calculated by Clapeyron equation (Equation (1)) ∆*V*_tr_ were determined for a 22-mer DNA duplex. For volumetric properties, the model that emphasizes the nature of the two bases in each dinucleotide step but does not distinguish the order (*i.e*., 5'-AC-3' and 5'-CA-3' are equivalent) was most appropriate. This trend can be explained if this property is dominated by the contribution of size of the dinucleotide step. Solvent accessible surface area, ∆*S*_A_, is widely used to characterize the surface size of a molecule accessible by solvent molecules [55,56,57,58]. Indeed, the ∆*S*_A_ values of each base pair revealed a good correlation with of *∆V*_tr_ values of the duplex (Figure 4b). Thus, kinetic analyses provide the activation volumes of the formation and melting of coil-to-helix transition of nucleic acids. By analysis of these parameters, it was concluded that hydrating water and interactions between nucleotide bases and sizes of bases contribute to annealing and melting reactions of nucleic acids.

### 2.5. Effect of Pressure on the Interactions between DNA and Protein

Reactions that occur along DNA (or RNA) such as replication, transcription, and recombination are carried out by numerous proteins and enzymes. During the recognition process between nucleic acid and protein electrostatic interactions, conformational changes, and hydration changes may occur. Therefore, it was hypothesized that pressure can regulate the interaction between protein and DNA, and that study of the effects of pressure will provide thermodynamic information on the reaction. Restriction endonucleases, which are an excellent model of DNA interacting proteins, have reduced ability to bind and hydrolyze DNA under high pressure, but the specificity of the reaction is enhanced [59,60]. High pressure may promote hydration of the enzyme and the enzyme-DNA interface [61], weakening non-specific interactions more than specific ones. LacI repressor protein adopts a tetrameric conformation that is destabilized in the presence of DNA at high pressure [62]; in contrast, dimerization of LexA repressor is stabilized upon DNA binding at high pressure due to effects of the condensation of each monomer on DNA [63]. Recently, the effects of pressure and temperature on the binding of RecA to a single-stranded DNA were investigated [64]. A phase diagram of ∆*G*(*P*, *T*) of formation of a RecA-DNA complex was obtained that indicated that the dissociation of the complex depended on the stability of RecA protein rather than DNA. This result agreed well with the structural analysis of DNA under high pressure described above [33,40]. Pressure can perturb the interaction between DNA and its cognate protein by changing the hydration in the protein, but there are no reports about the pressure perturbation to DNA-protein interaction due to the physical alterations of nucleic acid properties by pressure.

## 3. Non-Canonical Structures of Nucleic Acids under High Pressure

### 3.1. G-Quadruplex

The canonical structure of nucleic acids is a duplex stabilized by Watson-Crick base paring. Various non-canonical structures of nucleic acids have been identified and there is a growing body of evidence that these structures are adopted under certain conditions by genomic DNAs and transcribed RNAs in living cells. The G-quadruplex has received significant attention [65]; it is formed by stacking of guanine quartets (G-quartets), four guanine bases in a coplanar arrangement stabilized by Hoogsteen base pairing [66,67,68]. Although G-quadruplex structures are polymorphic depending on the sequence, metal ions, and the cosolute [69,70,71,72], all G-quadruplex structures have stacks of G-quartets and a central cavity that binds a monovalent cation, such as K^+^ or Na^+^, through interactions with the O6 carbonyls of the guanines [73]. The G-quadruplex can be intra- or intermolecularly, and exhibits a much more compact conformation than single-stranded nucleic acids [74]. Sequences with the potential to form G-quadruplex structures are located throughout the genome [75,76], and G-quadruplex structures appear to be involved in the regulation of gene expression, which includes not only telomere maintenance but also regulation of transcription, recombination, replication, and translation [14,77,78,79,80,81,82,83,84,85]. Key factors for the stabilization of G-quadruplexes are the incorporation of a monovalent cation, the number of G-quartets, and the lengths of loops [86,87], but the major force determining the stability of a G-quadruplex is hydration [88,89,90,91]. Unlike folding of a nucleic acid duplex, water molecules are released during the folding of G-quadruplex [90,91]. Therefore, volumetric analysis using high pressure has proven very useful for analysis of the mechanism of folding and unfolding of G-quadruplexes. 

**Figure 5:**
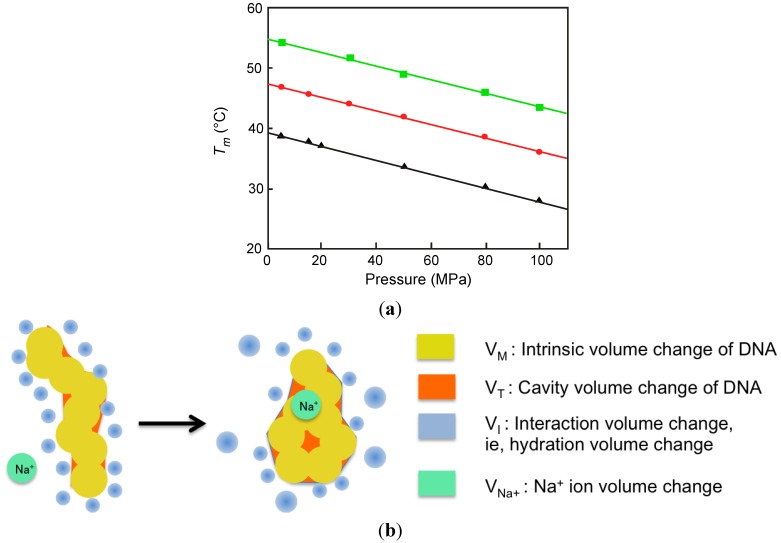
(**a**) Dependencies of the *T*_m_ for G-quadruplex DNA on pressure in the presence of 20 mM (▲), 50 mM (●), and 100 mM (■) NaCl [92]; (**b**) Graphical image of volumetric change of G-quadruplex based on Equation (4).

There are two excellent reports of the study of G-quadruplex structures under high pressure. The first was reported by Chalikian’s group [92]. This group studied the human telomeric (H-telo) oligonucleotide, d[A(GGGTTA)_3_GGG]. H-telo DNA forms a basket type G-quadruplex characterized by an antiparallel structure with one diagonal and two lateral loops [93]. The authors conducted UV melting under high pressure to monitor the unfolding process of H-telo oligonucleotide in the presence of Na^+^ ions. With increasing pressure, the melting temperature was remarkably decreased, indicating that *∆T*_m_/*∆P* was less than −10 × 10^−2^ K·MPa^−1^ (Figure 5a). 

Using the Clapeyron equation the transition volume, *∆V*_tr_, was determined to be 68 cm^3^·mol^−1^ in the presence of 20 mM Na^+^ ion. An increase in the concentration of NaCl to 100 mM was accompanied by a decrease of *∆V*_tr_ from 68 to 56 cm^3^·mol^−1^ (Table 3). These values indicate that the structure of the H-telo oligonucleotide is destabilized under high pressure, opposite of the thermostability of canonical duplexes, and that the change in volume is much larger than that of any other oligonucleotide structure characterized.

In order to estimate the contribution of the hydrating water in the transition of H-telo G-quadruplex, an approximate algorithm was presented. The change in volume associated with G-quadruplex formation *∆c*_tr_ determined experimentally can be summarized derived from Equation (2) as follows:

*∆V*_tr_ = *∆V*_M_ + *∆V*_T_ + *∆V*_I_ + *∆V*_Na+_ (4)

where *∆V*_M_ is intrinsic volume change of the DNA, *∆V*_T_ is thermal volume change indicating the change of the void space of the DNA, *∆V*_I_ is interaction volume change (*i.e.*, hydration volume change), and *∆V*_Na+_ is the volume change of incorporated sodium ion. By using molecular dynamics simulations of NMR structure of H-telo DNA (Figure 5b) [93], solvent-accessible surface area was estimated [56,57,58,59]. By using the empirical estimation of the thickness of the thermal volume and the known parameter of partial molar volume contribution of sodium ion [94,95,96,97], *∆V*_M_, *∆V*_T_, and *∆V*_Na+_ were estimated to be 233 cm^3^ mol^−1^, −370 cm^3^ mol^−1^, and −17.7 cm^3^ mol^−1^ (three Na^+^ ions), respectively. A *∆V*_tr_ value of 67 cm^3^ mol^−1^ was obtained from vibration tube densitometry. Therefore, *∆V*_I_ was estimated from Equation (3) as 186 cm^3^ mol^−1^ at 25 °C. *∆V*_I_ reflects water expansion around polar and charged groups of DNA during transition and is presented as:

*∆V*_I_ = *n*_h_ (*V*_h_ − *V*_0_) (5)

where *n*_h_ is the number of waters of hydration, *V*_h_ is the change in partial molar volume of water of hydration, and *V*_0_ is the change in bulk water. By using −1.8 cm^3^ mol^−1^ as the value of (*V*_h_ −*V*_0_) [98], a release of 103 water molecules occurs during the folding of H-telo DNA, which corresponds to about 18% of the net hydration of the coil formation. 

Another study from our group focused on the effect of molecular crowding conditions on G-quadruplex stability under high pressure [99]. Molecular crowding occurs in the presence of cosolute such as poly(ethylene glycol) (PEG) and mimics the conditions inside cells [100,101,102]. We have reported that crowding reagents like PEG stabilize G-quadruplexes due to changes in water activity and DNA hydration [90,103,104]. Chalikian’s group found that changes in hydration accompany the transition from coil to quadruplex [92]. It is possible that the volumetric characteristics of G-quadruplex DNA are also affected by molecular crowding agents.

**Table 3:** The value of the molar volume change ∆*V*_tr_ of the transition for G-quadruplex DNA.

DNA	Salt or Cosolute	∆*V*_tr_ (cm^3^ mol^−1^)
H-telo ^a^	[NaCl] = 20 mM	68 ± 2
	[NaCl] = 50 mM	60 ± 2
	[NaCl] = 100 mM	56 ± 2
TBA ^b^	(Absence)	54.6 ± 4.2
	40 wt% Ethylene glycol	12.5 ± 0.8
	40 wt% PEG 200	12.9 ± 0.9
	40 wt% PEG 4000	13.1 ± 1.0

^a^ Each solution was buffered with 10 mM sodium phosphate (pH 7.0), 0.1 mM EDTA, 0.1 mM NaN_3_, and each NaCl concentration [92]; ^b^ Each solution was buffered with 30 mM Tris-HCl (pH 7.0) and 100 mM KCl [100].

**Figure 6:**
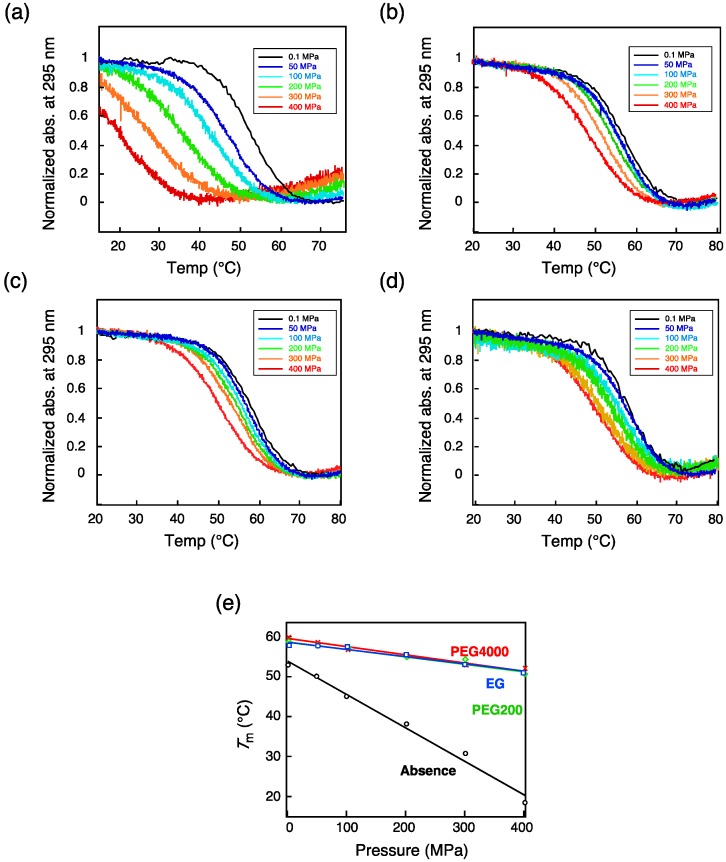
Effect of cosolute on the transition of 40 μM TBA from a quadruplex to a coil under various pressures [99]. UV melting curves were obtained (**a**) in the absence of cosolute or in the presence of (**b**) 40 wt% ethylene glycol, (**c**) 40 wt% PEG200, and (**d**) 40 wt% PEG4000. The changes of absorbance at 295 nm were analyzed under atmospheric pressure (0.1 MPa, black), 50 MPa (blue), 100 MPa (light blue), 200 MPa (green), 300 MPa (orange), and 400 MPa (red). Each solution was buffered with 30 mM Tris-HCl (pH 7.0) and contained 100 mM KCl. (**e**) Dependencies of the *T*_m_ for G-quadruplex DNA on pressure in the presence of ethylene glycol (blue), PEG200 (green), PEG4000 (red), and in the absence of cosolute (black).

In our study, we used the thrombin binding aptamer (TBA; 5′-GGTTGGTGTGGTTGG-3′) [105], which folds into an intramolecular, antiparallel G-quadruplex structure in the presence of various monovalent and divalent cations and cosolutes [88,90]. Temperature-dependent UV melting under high pressure was analyzed first in the presence of 100 mM KCl. In the absence of cosolute (PEG), the thermal stability was decreased with increasing pressure up to 400 MPa (Figure 6a) as observed for the H-telo DNA [92]. In contrast, in the presence of 40 wt% PEG, little unfolding of the TBA DNA was observed even under high pressure (Figure 6b–d). Our thermodynamic analysis indicated that crowding conditions repress the pressure effect due to enthalpic contributions. A volumetric analysis using the Clapeyron equation revealed that, in the absence of cosolute, *∆T*_m_/*∆P* was −8.4 × 10^−2^ K MPa^−1^ and *∆V*_tr_ was 54.6 cm^3^ mol^−1^, whereas in the presence of ethylene glycol, another crowding agent, *∆T*_m_/*∆P* was −1.9 × 10^−2^ K MPa^−1^ and *∆V*_tr_ was 12.5 cm^3^ mol^−1^ (Figure 6e, Table 3). PEG200 and PEG4000 (PEGs with average molecular weights of 200 and 4,000, respectively) caused effects similar to that of ethylene glycol (Table 3). We hypothesize that the crowding reagents did not affect the structure-dependent volume of TBA DNA and that *∆V*_M_, *∆V*_T_ and *∆V*_K+_ are the same in the absence or presence of crowding regents. Thus, *∆V*_I_ reflects the effect of high pressure in the presence of cosolute. Considering the tiny decrease of bulk water volume *V*_0_ in the presence of ethylene glycol or PEG [106,107], the Equation (5) indicates that the cosolute may decrease the number of hydration water (*n*_h_) and/or increase radii of hydrating waters to expand its volume (*V*_h_). Ethylene glycol or poly(ethylene glycols) decreases the volume change of the transition by one fourth due to the alteration of the number and/or radii of hydrating waters. The observed structural switching of DNA induced by pressure and cosolutes suggests that some gene expression may be regulated quadruplex by pressure changes in living cells.

### 3.2. Triple Helix

An oligonucleotide duplex can incorporate another strand via Hoogsteen base pairing to form a triple helix, also called a triplex. Wu and Macgregor examined the thermal stability of poly(dA)**·**poly(dT)_2_ under high pressure [23]. In the presence of 2 M NaCl, the triplex had a *∆T*_m_/*∆P* value of 4.50 × 10^−2^ K MPa^−1^ and relatively large magnitude *∆V*_tr_ (−7.81 cm^3^ mol^−1^). These parameters are obviously higher than those of poly(dA)**·**poly(dT) duplex. Thus, this result indicated that high pressure effectively stabilizes the triplex more than the duplex. An increase in the concentration of NaCl up to 3 M increased these parameters: *∆T*_m_/*∆P* was 5.80 × 10^−2^ K MPa^−1^ and *∆V*_tr_ was −10.4 cm^3^ mol^−1^. A kinetic analysis of the triplex formation was also reported [49]. The rate of *k*_−1_ for the unfolding process for the DNA triplex was affected by pressure more than was the rate of DNA duplex dissociation [48]. The activation volume for the triplex dissociation ∆*V*^‡^_−1_ was remarkably large at +39.9 cm^3^ mol^−1^ [48]. These values were determined in a different buffer than used for analysis of the duplex and so cannot be directly compared. 

### 3.3. Hairpin DNA

Hairpin DNAs or RNAs form intramolecularly and consist of a stem of Watson-Crick base pairs and a loop. The stability of a hairpin is determined by mainly by the sequence and number of base pairs within the stem region, but is affected by the loop sequence and by whether metal ions are bound [108,109,110,111]. Amiri and Macgregor investigated the stability of DNA hairpins under high pressure and determined volumetric parameters of hairpin DNAs containing different nucleation stacks and loop sequences. The systematic analysis revealed that the *∆V*_tr_ values of transition of coil-to-helix were as small as those of duplex but some *∆V*_tr_ values became positive at low sodium ion concentrations (Table 4). 

**Table 4:** Transition temperatures (*T*_m_) at atmospheric pressure and the molar volume change of the transition for the hairpin DNA with each loop sequence.

		Loop sequence					
		TA_2_T		TG_2_T		TC_2_T	
Nucleation stack	Na^+^ (mM)	*T*_m_ (°C)	∆*V*_tr_ (cm^3^ mol^−1^) ^a^	*T*_m_ (°C)	∆*V*_tr_ (cm^3^ mol^−1^)^ a^	*T*_m_ (°C)	∆*V*_tr_ (cm^3^ mol^−1^)^ a^
AT/AT	10	42.1	0.44	42.8	1.41	44.9	−1.81
	20	43.2	−0.18	44.0	0.25	46.1	−2.27
	50	44.6	−0.83	45.5	1.55	48.7	−3.05
	100	46.1	−1.46	46.8	−2.89	51.3	−3.76
AA/TT	10	40.2	1.96	37.9	2.35	44.0	−0.78
	20	41.5	1.15	41.1	0.86	45.4	−1.18
	50	43.3	0.19	43.5	−0.85	47.9	−1.75
	100	44.7	−0.74	45.1	−2.14	49.9	−2.35

^a^ The error was less than ± 0.32 cm^3^ mol^−1^.

For example, the DNA having TC_2_T loop with AA/TT nucleation stack (5′-GGATAATCCTTTAT CC-3′) had a negative *∆e*_tr_ value of −0.78 cm^3^ mol^−1^ in the each concentration of Na^+^ ion, whereas that with the TG_2_T loop with the same nucleation stack had a positive *∆V*_tr_ of 2.35 cm^3^ mol^−1^ in the presence of 10 mM Na^+^ ion. Considering that *∆V*_M_ in the equation (2) was negligible for DNA duplex [112,113], *∆V*_T_ and *∆V*_I_ are responsible for the contribution of each factor to *∆V*_tr_. Furthermore, *∆V*_T_, corresponding to the solvent accessible surface area *S*_A_ should be always negative because the coil form has a larger *S*_A_ than the helix. Therefore, *∆V*_I_ (hydration volume change) determines the magnitude of *∆V*_tr_, which in turn depends on the loop sequence and nucleation bases. For example, a loop consisting of purine bases had a positive volume change at low salt. These results imply that there are some specific interactions between the loop and cations. The importance of hydration within a loop region was also demonstrated by osmotic pressure analysis, which revealed that the loop region within a G-quadruplex determines the thermodynamic stability and hydration of the structure [114]. 

A simulation technique was also utilized to investigate the pressure effect on the folding/unfolding of the hairpin structure. Garcia and Paschek used replica exchange molecular dynamics (REMD) simulations to predict a pressure-temperature (*P*-*T*) free energy diagram for the RNA hairpin r(GCUUCGGC) and found that the RNA hairpin was destabilized by increases of pressure [115]. The change in volume was 4.1 cm^3^ mol^−1^, which was a relatively small change compared with that of the G-quadruplex. No other sequences of RNA or DNA have been studied by simulation techniques. 

## 4. Summary and Perspectives

In summary, we have reviewed papers related to the effect of pressure on nucleic acid structural conformations and stability. Pressure acts to compress the biomolecules. Molecular volume, compressivity, and expansibility depend on hydration and molecular packing, and the partial molar volume of a biomolecule can decrease or increase upon folding. The canonical DNA duplex formed with Watson-Crick base pairs generally has a negative partial molar volume of the melting transition (∆*V*_tr_), which indicates that applying pressure causes the duplex to be more stable. The typical magnitude of ∆*V*_tr_ for DNA duplexes is small compared with that of proteins. Only in specific cases such as poly[d(A-T)] do nucleic acid structures have a positive value of ∆*V*_tr_ and can melting induced by pressure change be observed. Structural analyses revealed that the conformation and configuration of DNA duplex are not significantly perturbed under high pressure. These results agree with studies of the interactions between proteins and DNA under high pressure in which it was observed that the conformation of the protein is only affected by pressure. 

In contrast to the stabilities of duplexes, which are relatively unaffected by pressure, non-canonical DNA (and RNA) structures are more sensitive to the pressure effect. G-quadruplex DNA structures are characterized by a positive and large ∆*V*_tr_ value, indicating that the G-quadruplex tends to unfold with increasing pressure and is much sensitive to pressure than the duplex form of DNA. The magnitude of the ∆*V*_tr_ value is generally 10 times greater than that of a duplex and more similar to magnitudes of ∆*V*_tr_ measured for proteins. Other DNA structures such as a triplex and a hairpin DNA have smaller changes in volume than do the G-quadruplexes but are more sensitive to pressure than a duplex. 

Osmotic pressure analysis show that DNA duplexes take up water molecules during the folding process [103,105], whereas G-quadruplexes and other non-canonical structures release water molecules [90,116,117]. The origin of different ∆*V*_tr_ between DNA duplex and these structures comes from the hydration. Interestingly, the number of water molecules taken up or released does not correspond to the difference of magnitude in change of ∆*V*_tr_ value. These results suggest that the physical properties of hydrating water around G-quadruplex are quite different from those of duplex. Further analysis for the hydration on non-canonical nucleic acids is needed.

Considering that G-quadruplexes and other non-canonical structures are sensitive to pressure changes, structural transitions induced by pressure may alter regulation of gene expression in cells. If local perturbations in pressure occur in cells, these changes may alter stabilities of duplex relative to non-canonical structures initiating or inhibiting cellular processes. As crowding conditions vary during the cell cycle [100], the stabilization of G-quadruplexes may depend on both cellular conditions and pressure. Recent study suggested that stress sensor protein Ras in human, which lives at atmospheric pressure, showed a relative small magnitude of transition volume of its reaction for the stress-signaling compared with those observed in G-quadruplexes [118]. And enzyme reaction such as replication and transcription may overcome the highly structured region of G-rich sequence with a help of relative low pressure, because some enzymes translocate along DNA with disrupting the proteins bound on DNA [119]. Therefore, the effect of pressure on quadruplex DNA in living cell may happen even at relative low pressure stress, at most 100 MPa, which is an acceptable pressure for living cells on earth. To discover genetic expression systems triggered by pressure is highly interesting and desired. 

Moreover, from the viewpoint of nanotechnology, DNA is a promising material for construction of sensors and nanostructures. In our previous paper [99], we utilized the property of quadruplex and duplex DNA to make switching DNA materials by pressure changes. It may be more and more possible to use pressure as a trigger to induce signals through structural changes in G-quadruplexes. 
